# Percutaneous autologus bone marrow injection in the treatment of delayed or nonunion

**DOI:** 10.4103/0019-5413.30529

**Published:** 2007

**Authors:** Rakesh Bhargava, SS Sankhla, Anil Gupta, RL Changani, KC Gagal

**Affiliations:** Department of Orthopedics, SMS Medical College, Jaipur, Rajasthan, India; * Department of Orthopedics, P S Medical College, Karamsad, Anand, Gujarat, India; ** Govt. Hospital, Alwar, Rajasthan, India

**Keywords:** Bone marrow injection, delayed union, nonunion

## Abstract

**Background:**

Bone marrow is a source of osteoprogenitor cells that are key elements in the process of bone formation and fracture healing. The purpose of the study was to ascertain the osteogenic potential of autologous bone marrow grafting and its effectiveness in the management of delayed union and nonunion.

**Materials and Methods:**

Twenty-eight patients with delayed union and three with nonunion of fracture of the long bones were treated with this procedure. Of these 28 cases, two patients had fracture shaft femur, one had fracture shaft ulna and 25 patients had tibial shaft fractures. The average time duration between procedure and injury was 25 weeks (range 14-53 weeks). The bone marrow was aspirated from the anterior iliac crest and injected percutaneously at the fracture site. The procedure was carried out as an outpatient procedure. All but five cases required one injection of bone marrow.

**Results:**

Union was observed in 23 cases. The average time of healing after the procedure was 12 weeks (range 7-18 weeks).

**Conclusion:**

The technique of percutaneous autologous bone marrow injection provides a very safe, easy and reliable alternative to open bone grafting, especially for early intervention in fracture healing process.

Despite continued improvement in operative fixation techniques, the method of autologous cancellous bone grafting to stimulate skeletal repair remained unchanged since the work of Phemister^1^ reported more than 50 years ago. Although it works in most of the cases but operative harvesting of the bone and implantation at fracture site has not been without complications. In addition to the donor site morbidity, the need to open the nonunion site has added the risk of infection or devascularization of the fracture fragments, where healing is already impaired. Perhaps this is why Boyd^2^ had said: “Bone grafting is primarily a second wounding procedure, in which surgeon hopes that the response of the body will be more favorable than the response following the original trauma.”

The search for a new osteoinductive substance has been the subject of great interest from the dawn of fracture management. Current research in basic science provides an understanding of the factors needed for osetogenesis in bone substitutes. The osteoblast is very well known as the chief bone-forming cell, but now it has been shown that osteoblasts, fibroblasts and reticular cells etc. have common precursor cells; and these common precursor cells are found in bone itself, in bone marrow and in certain areas of connective tissue framework.^3^–^6^

Cells aspirated from bone marrow are being shown to provide stimulus for osteogenesis in animal experiments and in clinical evaluation of bone graft and bone substitutes.^7^–^9^ Despite this osteogenic characteristic, the clinical use of marrow as an osteogenic source has remained limited. The marrow is harvested by needle aspiration from the patient's pelvic bone and is then injected percutaneously at the nonunion site. This method offers the advantage of treating fracture-healing problems without operative exposure of either the donor or recipient site.

The purpose of the present study is to ascertain the osteogenic potential of autologous bone marrow injection and its effectiveness in the management of delayed union and nonunion.

## MATERIALS AND METHODS

Twenty-eight patients of fracture of the long bones were treated with this procedure for delayed union (25) or nonunion (3). Of these 28 cases, two patients had fracture shaft femur, one had fracture shaft ulna and all other 25 patients had tibial shaft fracture. Six cases were closed fractures, 11 were Gustilo's Grade I, eight Grade II, two Grade IIIa and one Grade IIIb. Both femoral fractures were closed and treated by interlock nailing. Fracture ulna was compound Grade I and treated by IM square nail after debridement. Four cases with closed fracture shaft tibia were treated by close reduction and above knee cast application. In one open tibial fracture an IM “V” nail was put elsewhere, which got broken. We treated him conservatively with bone marrow injection and weight bearing in a PTB cast. Twelve cases with open fracture shaft tibia were treated by reduction and above knee cast application after surgical debridement. Four cases with open tibial fractures were put on external fixater initially, which was changed to either above knee cast or PTB cast after healing of the wound. One of these patients had gap nonunion following Grade IIIb tibial shaft fracture. In this patient fibula was grafted to fill the gap and marrow was injected to promote healing later on.

**Figure 1:**
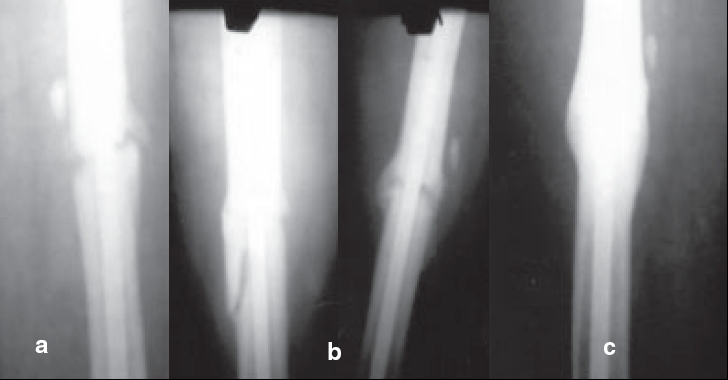
42 yrs. Female with, Close # Shaft femur (R) (a) 4 months after I/L nailing (before B.M. injection), (b) 6 weeks after B.M injection, (c) 18 weeks after B.M. injection

**Figure 2:**
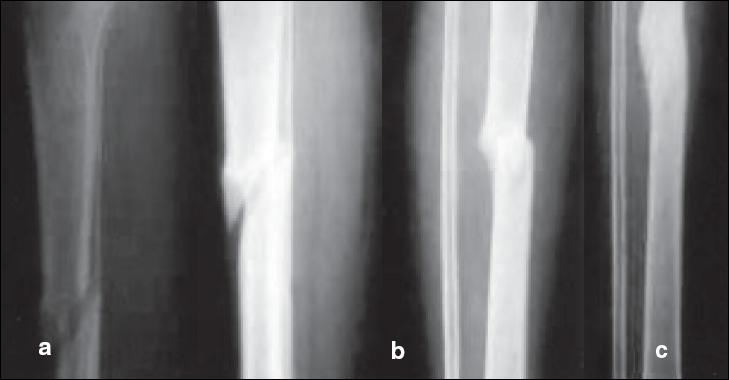
32 yrs. Female with Grade 1 Comp. # leg bones (R) (a)15 weeks after injury (before B.M. injection), (b) 9 weeks after B.M. injection, (c) 16 weeks after B.M. injection

Fractures with acceptable alignment and good bony apposition at fracture site were included in the study except for the case with gap nonunion and fibular grafting. After the initial procedure, partial weight bearing was allowed between 8-16 weeks as soon as the fracture became sticky. Tibial fractures were mobilized in a PTB cast while femoral fractures were mobilized after removing one set of locking screws. These patients were mobilized for a mean of 14.4 weeks (7-42 weeks) before being subjected to bone marrow injection. One patient with gap nonunion and fibular graft in tibia was never dynamized. The mean duration between injury and bone marrow injection was 25 weeks (range 14-53 weeks) [Figures 1 and 2]. In 25 cases of delayed union the duration was between 14 to 30 weeks, while in three cases of nonunion the duration was more then 40 weeks. One injection was found to be sufficient in the majority of cases, whereas only in five cases the procedure was repeated. Thus total 33 procedures were performed.

The standard procedure of aspiration of bone marrow from the anterior iliac blade and injecting percutaneously at fracture site was adopted. The procedure was carried out as an outpatient procedure. Twenty-three procedures were done under local infiltration with 2% xylocaine, nine procedures under short general anesthesia e.g. propofol or ketamine and one under spinal anesthesia. To aspirate the marrow from the iliac crest, a 16-gauge bone marrow aspiration needle was used and to inject the marrow at fracture site the standard disposable needle of 16-gauge in cases of tibia or ulna and a long 14/16-gauge abscess needle in case of femur was used. Image intensifier was used to locate the area of bone marrow injection. Only in a few cases of tibial fractures, the procedure was done without image intensifier as the fracture site was distinctly palpable. The volume of bone marrow injected was between 50-90 ml in cases of tibia and femur. In ulna the amount injected was 15 ml only. The limiting factor for the amount of bone marrow injection was the volume that could be injected at a particular site. The marrow was aspirated in 5-10 ml aliquots and injected at the fracture site simultaneously. Multiple entry portals were needed on one or both the iliac crests to harvest the marrow.

The postoperative management consists of a compression bandage for two or three days. After two or three days a well-fitting patellar tendon-bearing cast was applied in cases of fracture leg bones and patient was encouraged to walk. Two patients with fracture femur with IM nail were also encouraged to walk after the procedure. The patient with fracture shaft ulna was given a functional cast brace. The patients were followed up after every four weeks and the rate of healing was assessed clinically as well as radiologically. We used two scoring systems in this study.

### Union potential grading [Table 1]

**Table 1:** Union potential grading Max. score - 15; Excellent [E] - 13-15; Good [G] - 9-15; Poor [P] - <9

Features	1	2	3	4
Initial displacement	Complete displacement	Mild to moderate displacement	Undisplaced fractures	-
Communition	Severe communition >50%	Large butterfly segment	Mild or no communition	-
Soft tissue injury	Gustilo's type IIIB injury, where soft tissue cover couldn't be achieved satisfactorily within two weeks	Gustilo's type IIIA and IIIB injury, where soft tissue cover achieved satisfactorily within two weeks	Gustilo's type I and II injury	Close fractures
Wound infection	Moderate to severe infection taking > one wk. to settle down	Mild infection taking > one wk. to settle down	No infection at all
Alignment achieved	Poor reduction, coaptation <50% with angulation / rotation > 15 degree	Good reduction, coaptation >50%, with angulation/ rotation <15 degree	

Nicoll^10^ and Ellis^11^ investigated the factors leading to delayed and nonunion of tibial shaft fractures. Taking into account these common factors, we developed a scoring system to assess the chances of union in a particular fracture.

This score is based on the following five criteria:

1. Initial displacement. 2. Comminution. 3. Soft tissue injury, 4. Wound infection, 5. Alignment

The maximum score is 15. A score of 13 or more is graded as an excellent score and union in these cases can be predicted within the normal course of time. If the score is between 9 and 12, the chances of union are good but there are fair chances of delayed or even nonunion.

If the score is below 9, the union is unpredictable and there are high chances of delayed or nonunion without surgical intervention.

In our present study three cases were with excellent union potential score, 22 were with good and three cases were with poor union potential score.

### Union scale score [Table 2]

**Table 2:** Union scale score Max. score 7

Features	0	1	2	3
Mobility	Frank mobility in both planes	Restricted mobility in both planes	Minimum mobility in one plane	No mobility at all
Tenderness	Present	Absent	-	-
Radiological features	No callus at all	Minimum ensheathing callus	Good ensheathing callus or internal callus with bridging of at least two cortex	Good internal callus with bridging of all four cortex

To eliminate subjective bias in assessing bone union and to make comparisons more reliable, we developed this scoring system. This is a numerical score to assess the progress of union. The score has three criteria, namely fracture site mobility, tenderness and radiological fractures. A score of six or more was considered as sound union. In our present study union scale score at the time of first injection was zero in two cases, one in nine cases, two in 13 cases and three in one case.

Three cases, two fracture femur and one fracture ulna could not be assessed on this scale because of the IM nail *in situ*. The average union scale score at the time of first procedure was 1.52 (0-3).

## RESULTS

The union was achieved in 23 cases. Both the cases with fracture shaft femur and one case with fracture shaft ulna healed. All five cases of failure were fracture leg bones. All the three fractures with excellent union potential score, united. Nineteen out of 22 cases with good union potential score united, while union was achieved only in one case out of the three with poor union potential score. Of the three nonunion cases, union was achieved in two cases while the case with gap nonunion and fibular grafting failed to unite. Four out of five cases in which the procedure was repeated, healed.

The mean time of clinical and roentgenographic union was 12 weeks after injection (ranging from 7-18 weeks). The mean union scale score at the end of study was 5.39 (0-7) and in united cases it was 6.13 (6-7). No major complication was seen related to the procedure. Only a few patients (six out of 28) complained of dull ache at the donor site, which subsides in two to three weeks with reassurance and mild analgesics.

## DISCUSSION

Skeletal healing is primarily a biological process, and depends upon cellular response. The most productive source of cells that influence osteogenesis is considered to be autologus marrow. In the present study the effect of percutaneous autologous bone marrow injection in delayed union and nonunion of 28 long bones as a substitute for standard open grafting technique was studied.

The selection of cases included in the study had high selection bias. The majority of the cases (25 out of 28) were of delayed union. The bone marrow was injected in the majority of the cases (21 out of 28) within 30 weeks since injury. The majority of the cases (25 out of 28) had good or excellent union potential score. The reason was obvious. Initially, there was reluctance to use the procedure in patients with established nonunion presenting more than a year after the injury, with sclerosed bone ends, occluded marrow cavity or with features of bone infection. Hence the patients, who had good potential of healing according to the type of injury, but the fracture failed to unite in the expected time period, were chosen for the study. These were the cases who according to the union potential score had a fair chance of union but union didn't progress favorably and bone grafting was necessary to achieve union according to our usual management protocol. Such patients were advised percutaneous bone marrow injection instead of open bone grafting.

Although there was high selection bias in favor of union, it cannot be said that union in these cases would have occurred even without the procedure, as the mean time duration between procedure and injury was about 25 weeks, with average union scale score well below 2. A fair trial of dynamization in functional cast brace was given before embarking upon percutaneous bone marrow injection but the fracture failed to unite. After bone marrow injection the fractures united in mean 12 weeks. Hence it is clear that the percutaneous bone marrow injection had helped the fracture to unite. It had definitely accelerated the healing process. Although two out of three cases with established nonunion have united, the average time of healing was prolonged. It was 16 weeks as compared to an overall mean of 12 weeks. In one of these cases we had to repeat the procedure to achieve union. However the number is too small to reach any conclusion.

The work of Paley *et al*^12^ showed experimentally that marrow produces optimal effect when used early in the fracture healing process, with the poorest results encountered when used in the treatment of nonunion. Connolly *et al*^13^ also stated “Autologous bone marrow has been most useful for the preventive treatment of nonunion by early injection of delayed union’. He also said that the ideal time for bone marrow injection should be after the initial inflammatory and osteoclastic resorption period of fracture repair has subsided, which is usually by six to 12 weeks.^14^ So it can be inferred that the procedure should be done as soon as possible, when it is presumed that the union is not going to occur in the expected time duration. Although marrow by itself does not serve as an osteoconductive agent and therefore cannot fill a large gap, but it has been used by various investigators as composite graft with demineralized bone matrix,^8^,^9^,^15^,^16^ xenograft^17^ or porous ceramic materials^18^ in these situations. The use of marrow as composite graft with demineralized bone matrix for nontraumatic conditions like spinal fusions has also been described.^19^

No definitive conclusion can be made regarding the amount of marrow to be injected by this study as the volume of marrow injection was between 50-90 ml in all the cases of femur and tibia fractures. Different investigators have injected different amounts of bone marrow with good results but now it has been shown that efficacy of injected marrow is directly related to the number of progenitor cells.^9^

Healey^20^ in his study of eight cases injected around 50 ml of marrow. Four of them were injected only once, while another four were injected twice. The outcome was not significantly changed. Connolly *et al*^13^ injected around 100-150 ml of marrow in their cases. In all their 20 cases they used only one injection. Garg *et al*^21^ in their study injected 15-20 ml of bone marrow at the fracture site twice at three weekly intervals. It has also been shown that increasing concentration of marrow by centrifugation techniques increase its osteogenic activity.^7^,^9^ These techniques are extremely useful while using bone marrow where the fracture site is relatively small and space is limited as the volume of injection largely depends upon the site of injection.^7^

Heparin was not required in our study because of the short interval between aspiration and injection, thereby avoiding the potential impairment of bone healing associated with heparin reported by Stinchfield *et al*.^22^

## Conclusion

Autologous marrow grafting is a simple and effective method of providing cellular reactivation of osteogenesis without the complications and risks of cancellous bone grafting. The method can be used as an early intervention, whenever one suspects a delay in the healing of fracture. This procedure can be done on an outpatient basis and even under local anesthesia with minimal donor and recipient site morbidity.
